# lncRNA OIP5-AS1 attenuates the osteoarthritis progression in IL-1β-stimulated chondrocytes

**DOI:** 10.1515/med-2023-0721

**Published:** 2023-06-12

**Authors:** Xuefeng Zhang, Zhikun Wang, Binbin Wang, Jingyi Li, Hui Yuan

**Affiliations:** The First Clinical Medical College, Jinan University, Guangzhou, Guangdong 510632, P.R. China; Department of Pain, SSL Central Hospital of Dongguan, Affiliated Dongguan Shilong People’s Hospital of Southern Medical University, Dongguan, Guangdong 523326, P.R. China; Department of Orthopedics, SSL Central Hospital of Dongguan, Affiliated Dongguan Shilong People’s Hospital of Southern Medical University, Dongguan, Guangdong 523326, P.R. China

**Keywords:** osteoarthritis, OIP5-AS1, miR-338-3p, apoptosis, extracellular matrix degradation

## Abstract

In view of the association between long noncoding RNA OIP5-AS1 and osteoarthritis (OA) pathology, the corresponding potential mechanism is worthy of exploration. Primary chondrocytes were identified by morphological observation and immunohistochemical staining of collagen II. The association between OIP5-AS1 and miR-338-3p was analyzed by StarBase and dual-luciferase reporter assay. After the expression of OIP5-AS1 or miR-338-3p in interleukin (IL)-1β-stimulated primary chondrocytes and CHON-001 cells was manipulated, cell viability, proliferation, apoptosis rate, apoptosis-related protein (cleaved caspase-9, Bax) expressions, extracellular matrix (ECM) (matrix metalloproteinase (MMP)-3, MMP-13, aggrecan, and collagen II), PI3K/AKT pathway, and mRNA expressions of inflammatory factors (IL-6 and IL-8), OIP5-AS1, and miR-338-3p were determined by cell counting kit-8, EdU, flow cytometry, Western blot, and quantitative reverse transcription-polymerase chain reaction. As a result, the expression of OIP5-AS1 was downregulated in IL-1β-activated chondrocytes, while miR-338-3p was overexpressed. OIP5-AS1 overexpression reversed the effects of IL-1β on viability, proliferation, apoptosis, ECM degradation, and inflammation in chondrocytes. However, OIP5-AS1 knockdown exhibited opposite effects. Interestingly, the effects of OIP5-AS1 overexpression were partially offset by miR-338-3p overexpression. Furthermore, OIP5-AS1 overexpression blocked the PI3K/AKT pathway by modulating miR-338-3p expression. In sum, OIP5-AS1 promotes viability and proliferation, and inhibits apoptosis and ECM degradation in IL-1β-activated chondrocytes by targeting miR-338-3p through blocking the PI3K/AKT pathway, indicating an attractive strategy for OA treatment.

## 1 Introduction

Osteoarthritis (OA) is a type of joint disease, characterized by articular cartilage fibrosis, derangement, ulceration, and loss resulting from various factors [1]. As the aging of the population intensifies globally, OA is more prevalent and turns into a principal health problem in the world [2]. Nevertheless, OA progresses are still undefined, and a lack of efficacious options are provided to control or even reverse the course of OA [2]. Articular cartilage mainly comprises chondrocytes and extracellular matrix (ECM). The chondrocyte matrix is synthesized by chondrocytes that are necessary to maintain the normal structure and the function of the articular cartilage, and also experience many physiological changes in the OA stage, like proliferation and secretory profile [3]. Therefore, identifying the potential mechanism of chondrocyte dysfunction may shed new light on the progression of OA and contribute to discovering a novel effective method for the improvement of OA outcome.

Noncoding RNAs (ncRNAs) can be approximately divided into small (<200 nt) ncRNAs, covering microRNAs (miRNAs), and long ncRNAs (>200 nt, lncRNAs) [4]. Previous studies have indicated that the interaction between lncRNA and miRNA plays a critical part in OA pathogenesis [5,6,7]. Recent research showed that the downregulation of lncRNA OIP5-AS1 participates in the OA progression [8]. miR-338-3p is highly expressed in serum and cartilage tissues from knee OA patients, which thus can serve as a promising biomarker [9,10]. However, the effect and potential mechanism of miR-338-3p in chondrocyte dysfunction need to be explored. Moreover, Li et al. [11] reported that OIP5-AS1 functions as a ceRNA to regulate miR-338-3p expression.

Hypothetically, OIP5-AS1 may target miR-338-3p, thereby participating in the modulation of chondrocyte dysfunction. The current study sought to clarify the interaction between OIP5-AS1 and miR-338-3p in interleukin (IL)-1β-stimulated OA models using chondrocytes.

## 2 Materials and methods

### 2.1 Chondrocyte culture

Primary human chondrocytes were purchased from PromoCell (C-12710, Heidelberg, Germany). Cells were incubated in Dulbecco's Modified Eagle Medium (DMEM) (30-2002, ATCC, Manassas, VA, USA) with 10% fetal bovine serum (FBS; 16140071, Thermo Fisher Scientific) and 1% penicillin-streptomycin solution, and plated in a culture flask at 37°C with 5% CO_2_. Until the convergence rate reached 85%, the chondrocytes were digested and then passaged. The enzyme digestion method was the same as that in primary cells. The morphology of chondrocytes was observed with a microscope. The cells at P2/P3 passage were used in all experiments. Human chondrocyte cell line CHON-001 (CRL-2846) was procured from ATCC. CHON-001 cells were grown in DMEM containing 0.1 mg/mL G-418 (10131027, Gibco, Grand Island, NY) and 10% FBS and were incubated at 37℃ with 5% CO_2_.

### 2.2 Immunohistochemical staining

The expression of collagen II in primary chondrocytes at second, third, or fourth generation was observed using immunohistochemical staining. Following culture, the cells were smeared onto climbing slices, fixed in 4% formaldehyde (P0099, Beyotime, China) for 0.5 h, and permeabilized in 0.2% Triton X-100 (T8200, Solarbio) for 15  min. The slices were later washed with phosphate buffer saline (PBS) and then treated with 3% H_2_O_2_ (10011208, Sinopharm Chemical Reagent Co. Ltd., Shanghai, China) for 15 min to repress endogenous peroxidase. After that, the cells were incubated with rabbit anti-Collagen II primary antibody (ab34712, 1/200 dilution, Abcam, Cambridge, MA, USA) overnight at 4°C, followed by treatment with horseradish peroxidase (HRP)-conjugated secondary antibody (ab205718, 1/20,000 dilution, Abcam) for 1 h at room temperature. Diaminobenzidine (DAB, ab103723, 1/100 dilution, Abcam) was used as the chromogen to stain the cells, and then the cells were counterstained with hematoxylin (H8070, Solarbio). The images were captured using a microscope (E800, Nikon, Tokyo, Japan) under ×200 magnification (Scale bars, 20 μm). Positive expression was presented as yellow or brown staining.

### 2.3 Cell transfection

An overexpression vector (pcDNA3.1; V79520, Invitrogen™, Thermo Fisher Scientific) containing human OIP5-AS1 gene (NCBI Accession Number: NR_026757.2) was generated by RiboBio (Guangzhou, China), with pcDNA3.1 empty vector as the negative control. The siRNA against OIP5-AS1 (si-OIP5-AS1; siB170717102527-1-5) and siRNA negative control (siNC; A06001) were synthesized by GenePharma (Shanghai, China). miR-338-3p mimic (M; miR10000763-1-5) and its negative control (mimic control (MC); miR1N0000001-1-5) were purchased from RiboBio (Guangzhou, China). Chondrocytes were plated into six-well plates until 60% confluence was reached and then transfected with oligonucleotides and/or plasmids with lipofectamine 3000 reagent (L3000008, Thermo Fisher Scientific) for 48 h.

### 2.4 StarBase analysis and dual-luciferase reporter assay

The binding sites between miR-338-3p and OIP5-AS1 were predicted by StarBase 3.0 software. A pmirGLO vector (E1330, Promega, USA) containing wild-type (WT) or mutant (MUT) OIP5-AS1 3′-UTR was constructed. HEK293 cells (CRL-1573, ATCC, Manassas, VA, USA) were incubated in 24-well plates overnight. Next, cells were co-transfected with OIP5-AS1-WT vectors/OIP5-AS1-MUT vectors and miR-338-3p mimics/MC for 48 h. Thereafter, the luciferase activity was detected by the dual-luciferase reporter system (E1910, Promega) under a multifunction microplate reader (GENios-Pro 96/384, Tecan, Milan, Italy).

### 2.5 OA model establishment and grouping of chondrocytes

An *in vitro* OA model of chondrocytes was established by the stimulation of 10 ng/mL IL-1β (SRP3083, Sigma-Aldrich, Shanghai, China) for 24 h [12]. To identify the effect of OIP5-AS1 on OA, six groups were designed as follows: control, IL-1β, IL-1β + vector, IL-1β + OIP5-AS1, IL-1β + siNC, and IL-1β + siOIP5-AS1. Following transfection of OIP5-AS1 overexpression plasmid, siOIP5-AS1, or empty vector, an OA model of chondrocytes was established with IL-1β stimulation. Cells cultured in a medium without transfection and IL-1β stimulation served as the control group.

Furthermore, to identify whether miR-338-3p modulates the function of OIP5-AS1 in OA progression, four groups were designed as follows: IL-1β + vector + MC, IL-1β + OIP5-AS1 + MC, IL-1β + OIP5-AS1 + M, and IL-1β + vector + M. Before stimulation with 10 ng/mL IL-1β for 24 h, chondrocytes were transfected with MC/miR-338-3p mimic and empty vector/OIP5-AS1.

### 2.6 Cell counting kit-8 (CCK-8) assay

In short, 100 μL transfected or nontransfected chondrocytes were placed into 96-well plates (1 × 10^4^ cells/well) and subsequently stimulated with 10 ng/mL IL-1β for 24 h. Then, the cell viability was assessed using 10 μL CCK-8 solution (C0038, Beyotime, Shanghai, China), and the optical density at 450 nm was evaluated using a multifunction microplate reader.

### 2.7 EdU assay for detecting cell proliferation

After transfection, the cells were seeded into 96-well plates (4 × 10^3^/well). The cell proliferation was measured by EdU assay according to the manufacturer’s protocol. Following 2 h of incubation with 100 μL EdU solution (50 μM, R1053.11, RibBio, Guangzho, China) containing medium, the cells were fixed with 50 μL of 4% paraformaldehyde for 30 min at 37℃. Then, the fixed cells were incubated with 50 μL glycine (2 mg/mL) on a decolorizing shaking bed for 5 min, after the fixative was discarded, followed by washing with PBS and permeabilization with 100 μL Triton X-100 (0.5%). After EdU labeling, the cells were treated with 100 μL of Apollo reaction cocktail, and cell nuclei were stained with Hoechst 33342 (100 μL). Finally, EdU-positive cells were observed under a fluorescence microscope and quantified using Image J.

### 2.8 Flow cytometry assay

Briefly, transfected or nontransfected chondrocytes were inoculated into 24-well plates (5 × 10^5^ cells/well) and then treated with IL-1β for 24 h. Later, the apoptotic rate of chondrocytes was assessed with Annexin V-FITC/PI apoptosis detection kit (CA1020, Solarbio) using a flow cytometry instrument (BD Accuri C6 flow cytometer).

### 2.9 Quantitative reverse transcription-polymerase chain reaction (qRT-PCR)

The total RNAs were extracted using TRIzol (12183555, Thermo Fisher Scientific). PrimeScript™ RT reagent Kit (RR037A, Takara, Dalian, China) was utilized to obtain cDNA from 1 μg of total RNA, with random primers and oligo dT. The miRNA was reversely transcribed into cDNA using PrimeScript™ RT reagent kit, with stem-loop RT primers (listed in Table 1). The cDNA was amplified using TB Green^®^ Premix Ex Taq™ II (RR820A, Takara) on Thermal Cycler Dice™ Real Time System (TP700, Takara). Relative expression levels of genes were calculated based on the 2^−ΔΔCt^ method [13]. GAPDH or U6 was the internal control for mRNA or miRNA, respectively. The primer sequences were included in Table 1.

**Table 1:** Primer sequences used in qRT-PCR

Gene	RT (5′ → 3′)	Forward (5′ → 3′)	Reverse (5′ → 3′)
miR-338-3p	GTCGTATCCAGTGCGTGTCGTGGAGTCGGCAATTGCACTGGATACGACCAACAAAA	GTATCCAGTGCGTGTCGTGG	TGTTGGTCGTATCCAGTGCAA
U6	CGCTTCACGAATTTGCGTGTCAT	CGCTTCACGATTTGCGTGTCAT	GCTTCGGCAGCACATATACTAAAAT
OIP5-AS1	Random hexamer	GGCUGAGUUUCAUUUGAAACAGGTG	CACCUGUUCAAAUGAAACUCAGCCUU
IL-6	Random hexamer	ACTCACCTCTTCAGAACGAATTG	CCATCTTTGGAAGGTTCAGGTTG
IL-8	Random hexamer	TTTTGCCAAGGAGTGCTAAAGA	AACCCTCTGCACCCAGTTTTC
GAPDH	Random hexamer	CACCACACTGAATCTCCCCT	TGGTTGAGCACAGGGTACTT

### 2.10 Western blot

The total protein was lysed from cells in radio immuno precipitation assay buffer (R0010, Solarbio) with protease and phosphatase inhibitor (P1045, Beyotime, 1:50) for 30 min at 4°C, and then quantified with a BCA kit (PC0020, Solarbio). ColorMixed Protein Marker (PR1920, Solarbio) served as a protein size marker. Then, protein (20 μg) was isolated by 6–10% sodium dodecyl sulphate-polyacrylamide gel electrophoresis gels (P1200, Solarbio) and transferred onto immobilon-P polyvinylidene fluoride membranes (YA1701, Solarbio). Following incubation with 5% bovine serum albumin (SW3015, Solarbio) for 1 h at room temperature, the membranes were treated with primary antibodies (shown in Table 2) at 4℃ overnight, and then corresponding secondary antibody (shown in Table 2) for 2 h at room temperature. Visualization was achieved with ECL Western Blotting Substrate (PE0010, Solarbio). The intensity of protein band was determined by Quantity One Analysis Software (version 4.62; Bio-Rad Laboratories, Inc.).

**Table 2:** List of antibodies used for western blots

Protein	Host species	Catalog number	Company	Antibody dilution
Cleaved caspase-9	Rabbit	#9505	CST	1:1,000
Bax	Rabbit	ab32503	Abcam	1:1,000
MMP-3	Rabbit	ab52915	Abcam	1:1,000
MMP-13	Rabbit	ab39012	Abcam	1:6,000
Aggrecan	Mouse	ab3778	Abcam	1:1,000
Collagen Ⅱ	Rabbit	ab188570	Abcam	1:1,000
PI3K	Rabbit	#4292	CST	1:1,000
p-PI3K	Rabbit	ab182651	Abcam	1:500
Akt	Rabbit	ab8805	Abcam	1:500
p-Akt	Rabbit	ab38449	Abcam	1:500
β-actin	Mouse	ab8226	Abcam	1:1,000
Secondary antibody	Goat Anti-Rabbit IgG H&L (HRP)	ab205718	Abcam	1:2,000
Secondary antibody	Goat Anti-Mouse IgG H&L (HRP)	ab205719	Abcam	1:2,000

### 2.11 Data analysis

Data were analyzed using GraphPad Prism 8.0 (San Diego, CA, USA) and shown as the mean ± standard deviation (SD, *n* = 3). The independent sample *t*-test was utilized to compare the differences between the two groups. Comparisons of multiple groups were completed using analysis of variance. Bonferroni test was employed for Post hoc pairwise comparison. *p* < 0.05 was regarded as a statistically significant difference.

## 3 Results

### 3.1 Identification of primary chondrocytes and downregulation of OIP5-AS1 in OA chondrocytes

**Figure 1:**
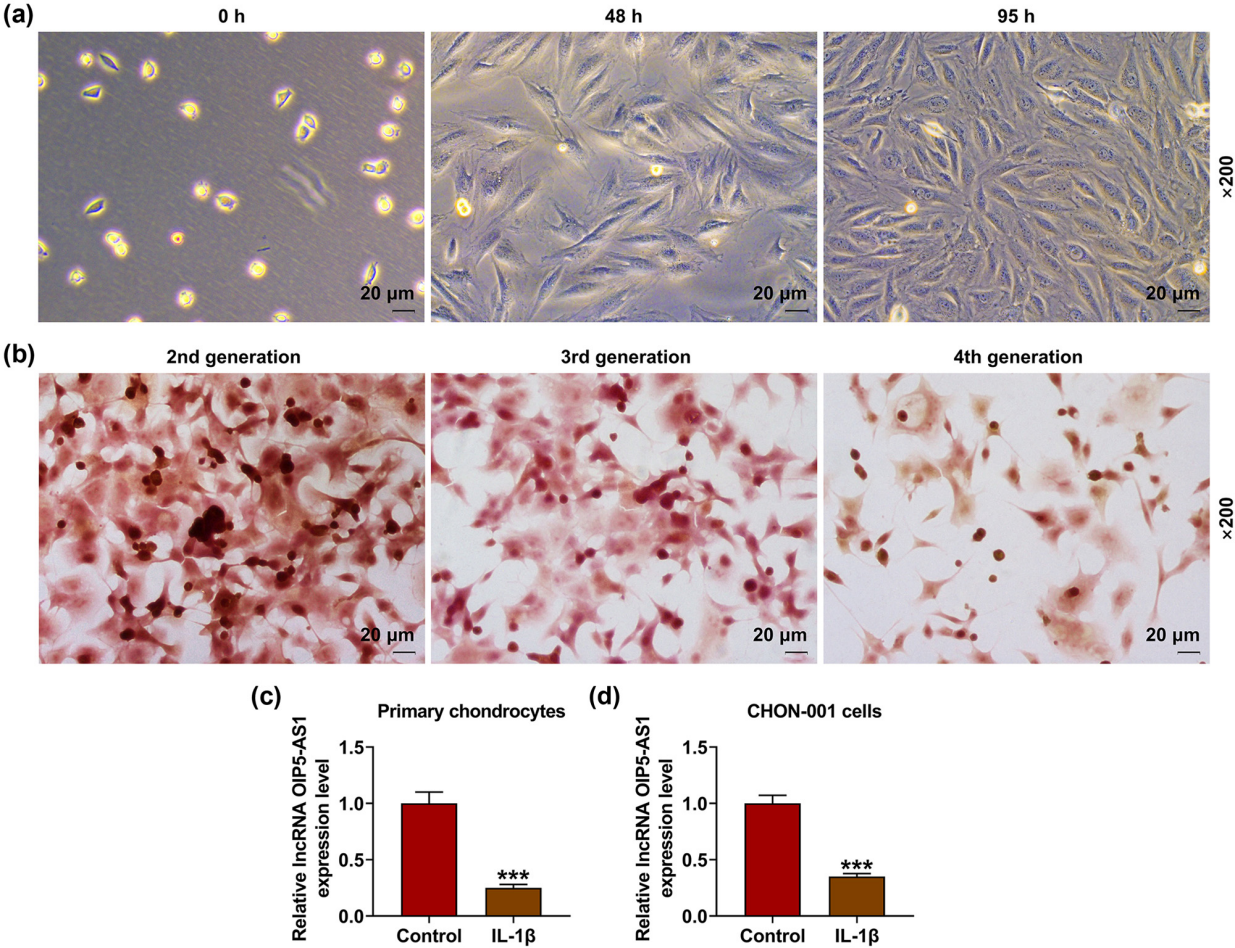
Identification of primary chondrocytes and downregulation of OIP5-AS1 in OA chondrocytes. (a and b) Human primary chondrocytes were identified by morphological observation (a) and immunohistochemical staining of collagen II (b). (c and d) The level of OIP5-AS1 in IL-1β-stimulated primary chondrocytes and CHON-001 cells was determined by qRT-PCR. IL-1β, interleukin-1β; OA, osteoarthritis; qRT-PCR, quantitative reverse transcription-polymerase chain reaction. ^***^ *p* < 0.001 vs control.

As shown in Figure 1a, under the inverted microscope, the primary chondrocytes were observed to be spherical, homogenous in size, and not easy to adhere to the wall. Forty-eight hours after incubation, the cells were oval or spindle like, more adhesive to the wall, and rich in cytoplasm, and cell nuclei presented an elongated morphology. Ninety-five hours after incubation, cells grew rapidly, and spindle-like cells were observed to further proliferate, whereas part of the cells gradually became rectangular or polygonal; moreover, cells began to connect with each other, grown in colonies, adhered to the wall, and covered the bottom of the flask. According to Figure 1b, cells at passage 2 exhibited good coloring; coloring in cells at passage 3 was waning, but still present; and cells at passage 4 exhibited poor coloring and had a greater variation. Therefore, chondrocytes at passage 2 and 3 were used for the subsequent experiments. In addition, the level of OIP5-AS1 was downregulated in IL-1β-activated chondrocytes (Figure 1c and d, *p* < 0.001). These findings indicated the downregulation of OIP5-AS1 in IL-1β-stimulated chondrocytes.

### 3.2 OIP5-AS1 regulated viability, proliferation, and apoptosis of IL-1β-activated chondrocytes

**Figure 2:**
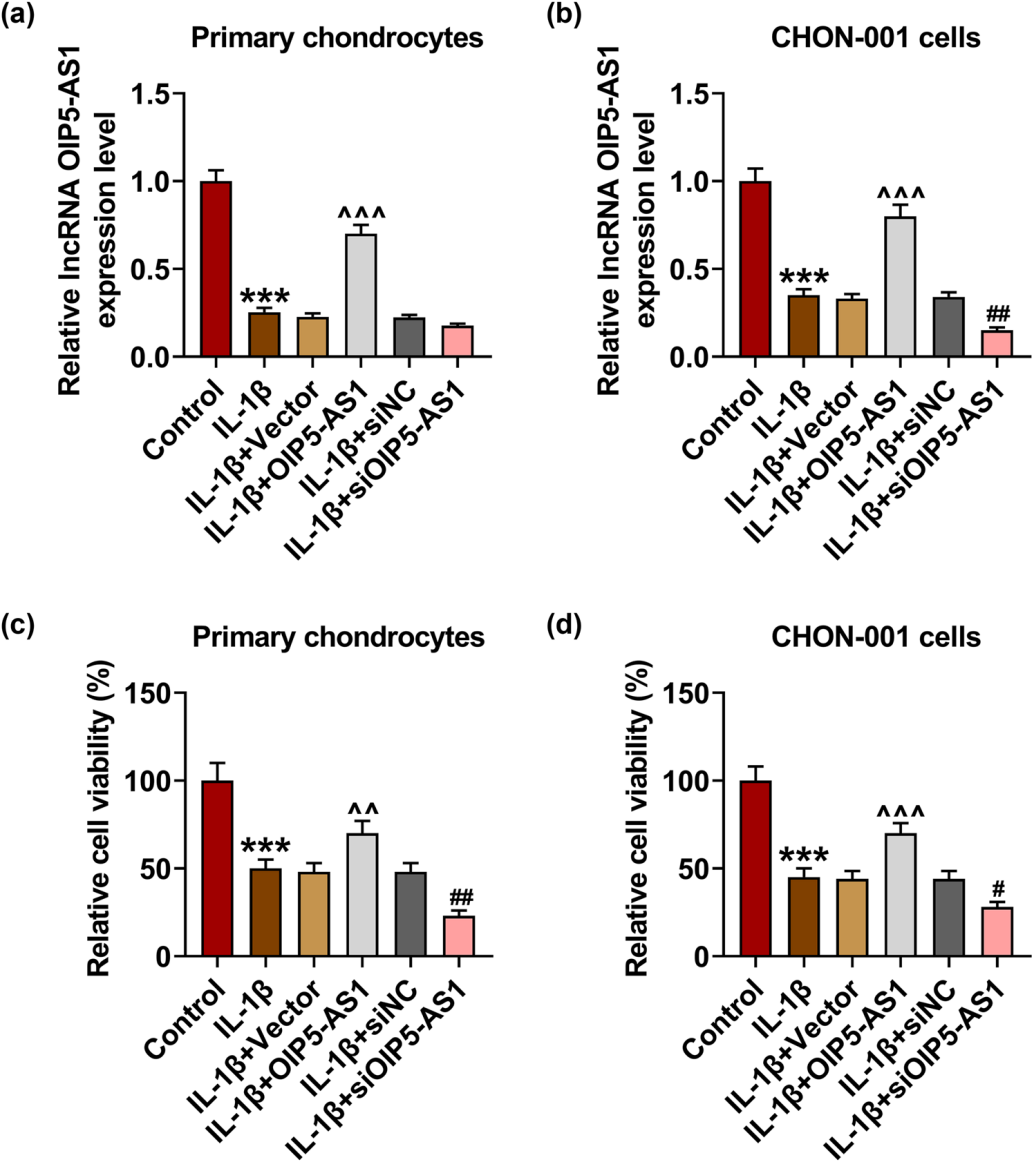
OIP5-AS1 regulated viability of IL-1β-activated chondrocytes. (a and b) The level of OIP5-AS1 in chondrocytes was detected using qRT-PCR, with GAPDH as internal control. (c and d) Transfected chondrocytes were subjected to PBS or IL-1β, and then cell viability was examined by cell counting kit-8 (CCK-8). IL-1β, interleukin-1β; PBS, phosphate buffer saline. ^***^ *p* < 0.001 vs control; ^^^^ *p* < 0.01, ^^^^^ *p* < 0.001 vs IL-1β + vector; ^#^ *p* < 0.05, ^##^ *p* < 0.01 vs IL-1β + siNC.

**Figure 3:**
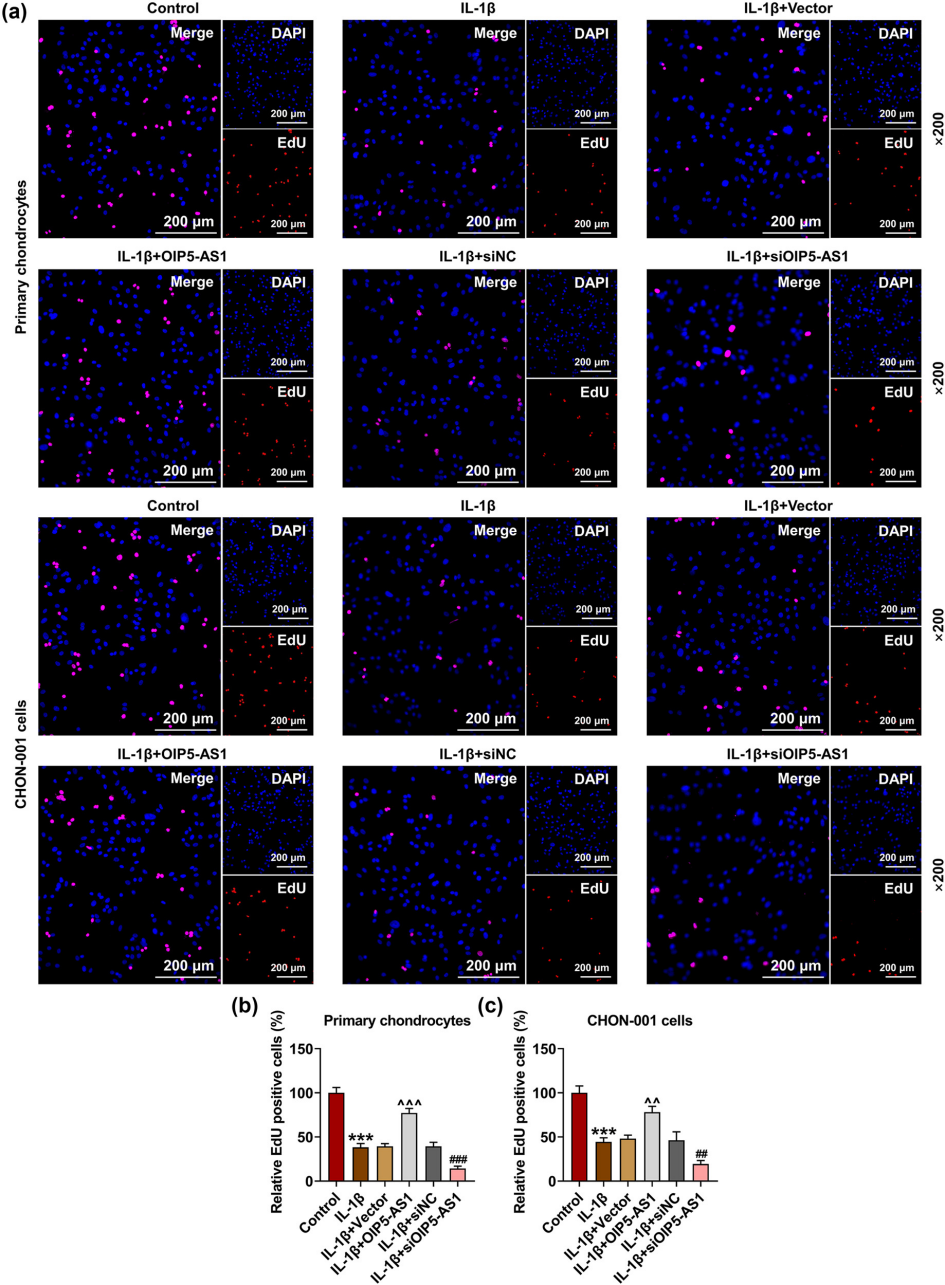
OIP5-AS1 regulated proliferation of IL-1β-activated chondrocytes. (a–c) Transfected chondrocytes were subjected to PBS or IL-1β, and then cell proliferation was examined by EdU assay. ^***^ *p* < 0.001 vs control; ^^^^ *p* < 0.01, ^^^^^ *p* < 0.001 vs IL-1β + vector; ^##^ *p* < 0.01, ^###^ *p* < 0.001 vs IL-1β + siNC. IL-1β, interleukin-1β; PBS, phosphate buffer saline.

**Figure 4:**
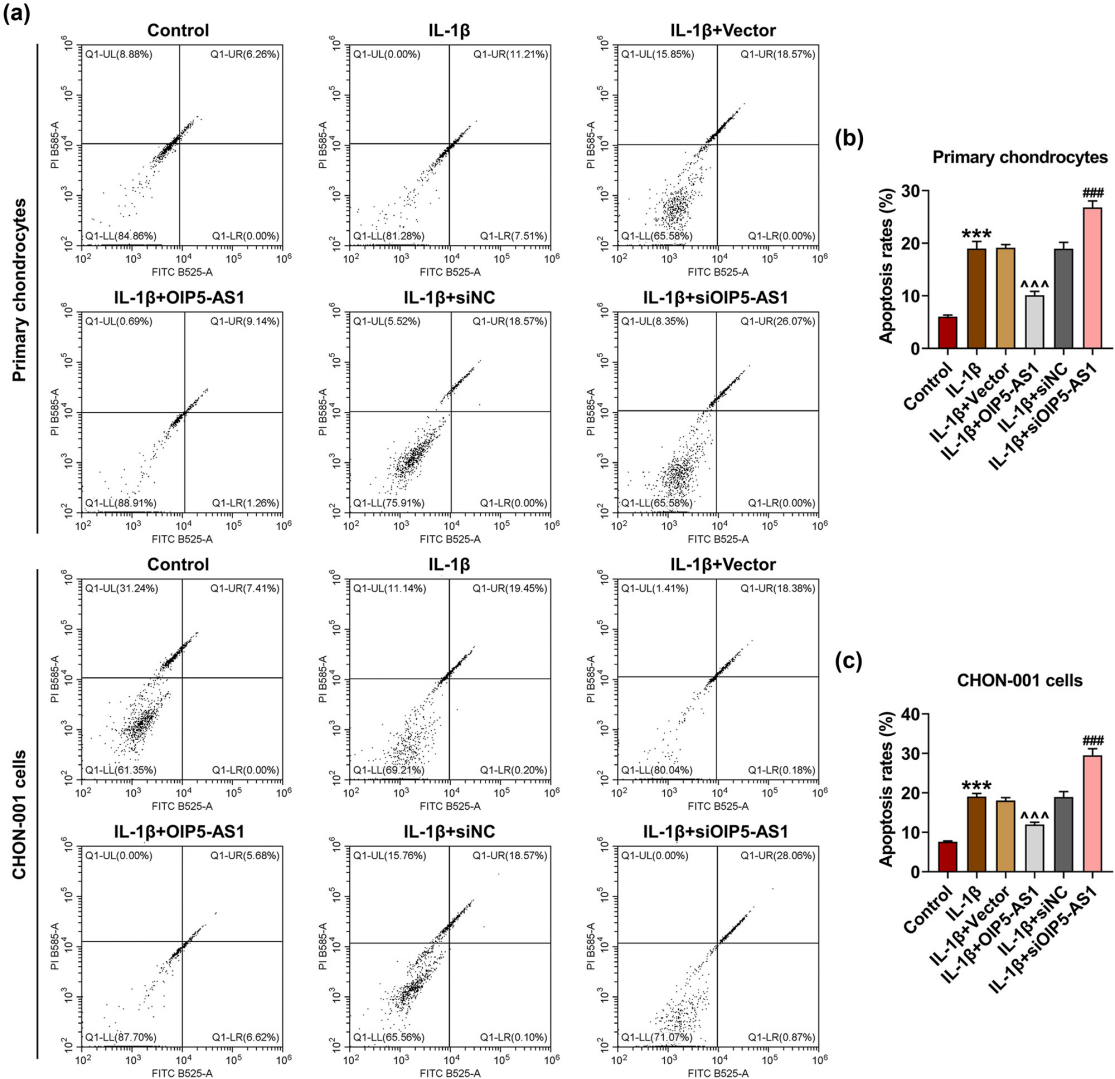
OIP5-AS1 modulated apoptosis rate of IL-1β-activated chondrocytes. (a–c) Transfected chondrocytes were subjected to PBS or IL-1β, and then cell apoptosis was detected using annexin V-FITC/PI staining. IL-1β, interleukin-1β; PBS, phosphate buffer saline. ^***^ *p* < 0.001 vs control; ^^^^^ *p* < 0.001 vs IL-1β + vector; ^###^ *p* < 0.001 vs IL-1β + siNC.

**Figure 5:**
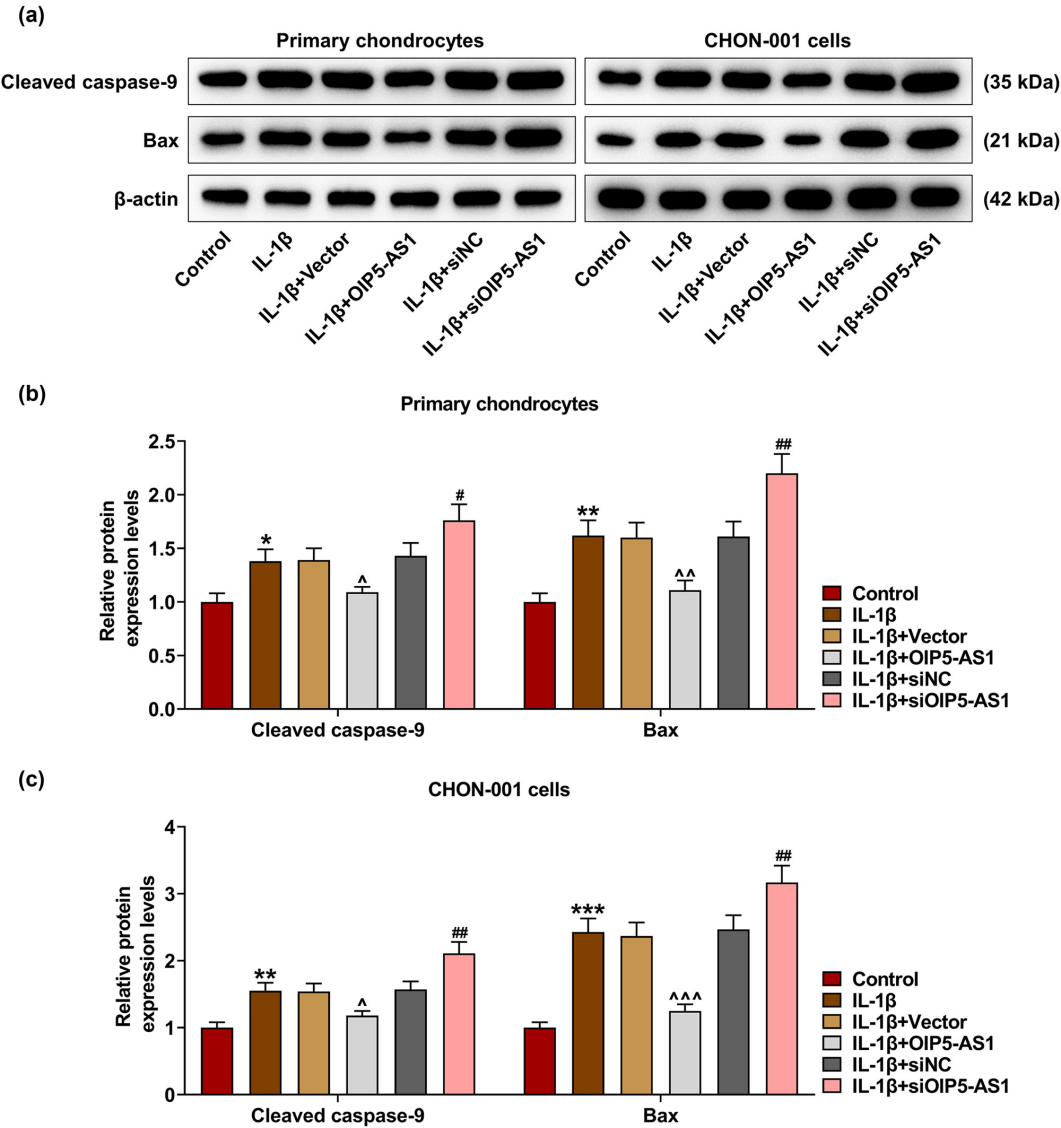
OIP5-AS1 modulated apoptosis-related proteins of IL-1β-activated chondrocytes. (a–c) The protein levels of cleaved caspase-9 and Bax were measured using Western blot. ^*^ *p* < 0.05, ^**^ *p* < 0.01, ^***^ *p* < 0.001 vs control; ^^^ *p* < 0.05, ^^^^ *p* < 0.01, ^^^^^ *p* < 0.001 vs IL-1β + vector; ^#^ *p* < 0.05, ^##^ *p* < 0.01 vs IL-1β + siNC.

When compared to IL-1β + vector group, in the IL-1β + OIP5-AS1 group, the level of OIP5-AS1 was elevated in IL-1β-activated chondrocytes, while in IL-1β + siOIP5-AS1 group, the level of OIP5-AS1 was decreased (Figure 2a and b, *p* < 0.001). It was found that IL-1β stimulation strikingly inhibited cell viability (Figure 2c and d, *p* < 0.001), proliferation (Figure 3a–c, *p* < 0.001), and enhanced apoptosis rate (Figure 4a–c, *p* < 0.001) of chondrocytes, which was reversed by OIP5-AS1 overexpression (Figures 2c, d, 3a–c, and 4a–c, *p* < 0.001), but was further strengthened by OIP5-AS1 knockdown (Figures 2c and d, 3a–c, and 4a–c, *p* < 0.05). Apoptosis regulators like caspases and Bcl-2-associated X protein (Bax) are critical execution enzymes in mitochondria-mediated apoptosis [14]. In this study, IL-1β stimulation upregulated the expressions of cleaved caspase-9 and Bax in chondrocytes (Figure 5a–c, *p* < 0.05), which was offset by OIP5-AS1 overexpression (Figure 5a–c, *p* < 0.05), but was further promoted by OIP5-AS1 silencing (Figure 5a–c, *p* < 0.01). The aforementioned findings implied that OIP5-AS1 regulated viability, proliferation, and apoptosis of IL-1β-activated chondrocytes.

### 3.3 OIP5-AS1 modulated ECM degradation and inflammation of IL-1β-activated chondrocytes

**Figure 6:**
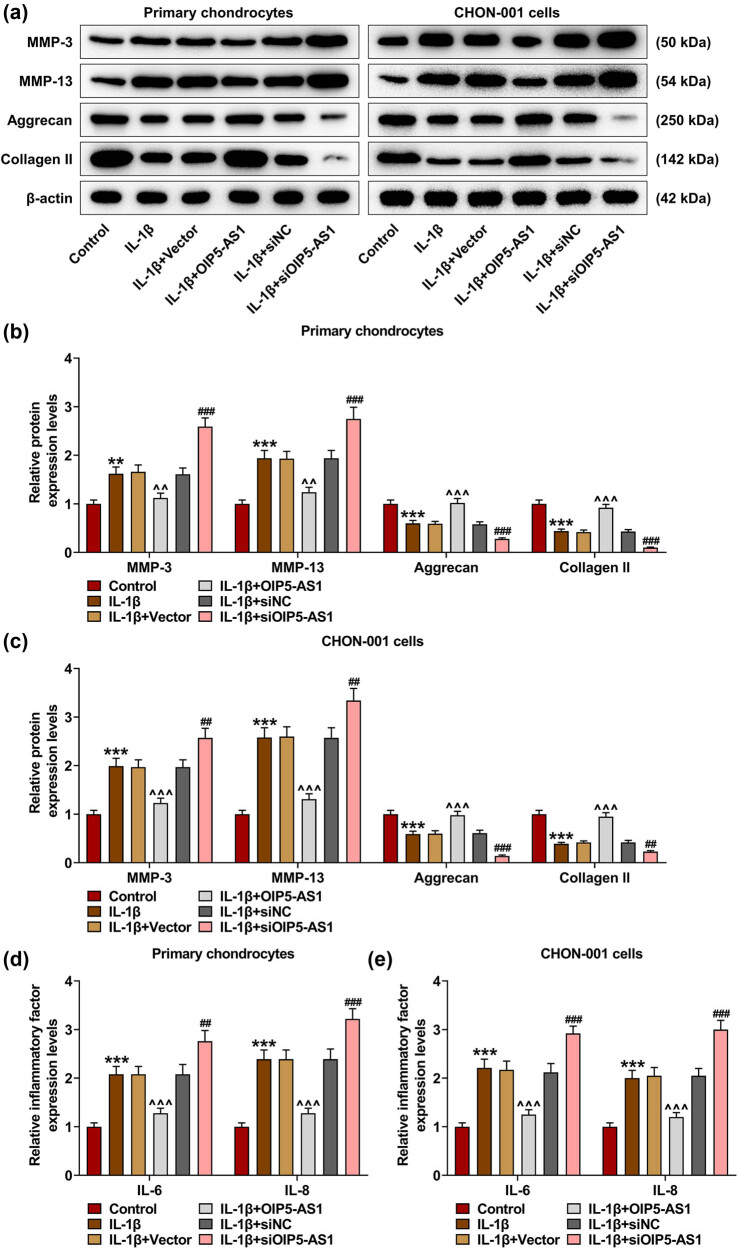
OIP5-AS1 mediated ECM degradation and inflammation of IL-1β-activated chondrocytes. (a–e) Transfected chondrocytes were subjected to PBS or IL-1β. The protein levels of MMP-3, MMP-13, aggrecan and collagen II were quantified using Western blot (a–c), and the mRNA levels of inflammatory factors IL-6 and IL-8 were measured by qRT-PCR (d and e). IL-1β, interleukin-1β; MMP, matrix metalloproteinase; PBS, phosphate buffer saline; qRT-PCR, quantitative reverse transcription-polymerase chain reaction. ^**^ *p* < 0.01, ^***^ *p* < 0.001 vs Control; ^^^^ *p* < 0.01, ^^^^^ *p* < 0.001 vs IL-1β + Vector^; ##^ *p* < 0.01 vs IL-1β + siNC.

ECM synthesized by chondrocytes is necessary to maintain the normal structure and the function of the articular cartilage. In the stage of OA, matrix metalloproteinase (MMP)-3 and MMP-13 are indispensable for cartilage degradation [15]. Aggrecan is also the main structural ingredient of ECM. In addition, MMP-13 is regarded as a primary contributor to cartilage degeneration of OA because it preferentially cleaves collagen II [16]. In line with Figure 6a–c, IL-1β augmented the levels of MMP-3 and MMP-13, while diminishing the levels of aggrecan and collagen II in chondrocytes (*p* < 0.01). Also, the effects of IL-1β stimulation on these genes were partially mitigated by OIP5-AS1 overexpression (*p* < 0.01), but were potentiated by OIP5-AS1 knockdown (*p* < 0.01). In addition, IL-1β stimulation elevated the levels of IL-6 and IL-8 in chondrocytes (Figure 6d and e, *p* < 0.001), which was offset by OIP5-AS1 overexpression (Figure 6d and e, *p* < 0.001), but was further promoted by OIP5-AS1 knockdown (Figure 6d and e, *p* < 0.01). These data manifested that OIP5-AS1 regulated ECM degradation and inflammation of IL-1β-activated chondrocytes.

### 3.4 miR-338-3p was inversely modulated by OIP5-AS1 and overexpressed in IL-1β-activated chondrocytes

**Figure 7:**
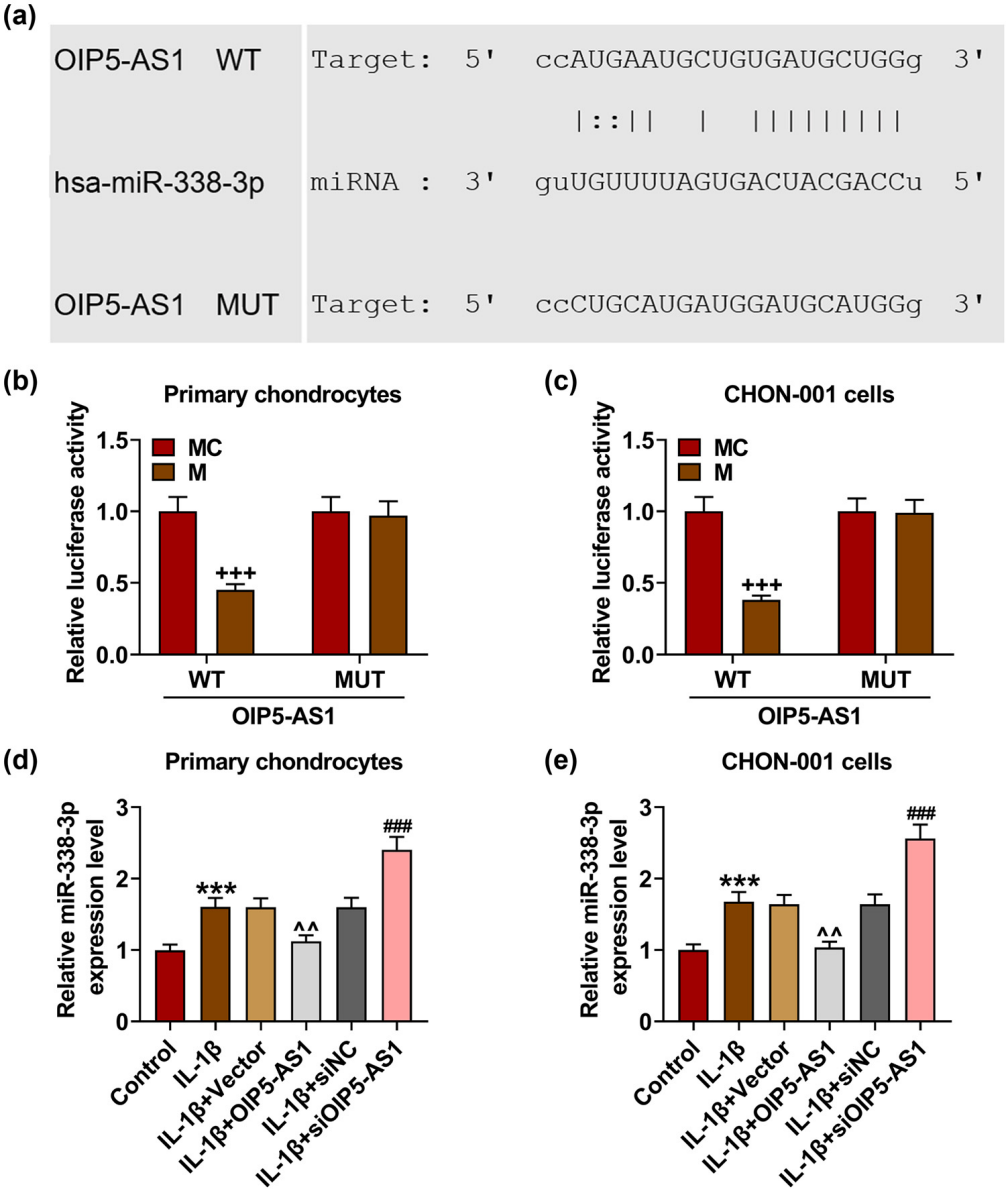
MiR-338-3p was negatively regulated by OIP5-AS1 and was overexpressed in IL-1β-activated chonarocytes. (a) The targeting relationship between miR-338-3p and OIP5-AS1 was analyzed with StarBase. (b and c) The luciferase activity was examined. (d and e) qRT-PCR was performed to detect the level of miR-338-3p in IL-1β-stimulated chondrocytes, with U6 as internal control. IL-1β, interleukin-1β; WT, wild-type; MUT, mutant; qRT-PCR, quantitative reverse transcription-polymerase chain reaction. ^+++^ *p* < 0.001 vs MC; ^***^ *p* < 0.001 vs Control; ^^^^ *p* < 0.01 vs IL-1β + Vector; ^###^ *p* < 0.001 vs IL-1β + siNC.

The binding relation between miR-338-3p and OIP5-AS1 was shown by StarBase (Figure 7a) and verified by the results from dual-luciferase reporter assay (Figure 7b and c, *p* < 0.001). In addition, IL-1β stimulation promoted miR-338-3p expression in chondrocytes (Figure 7d and e, *p* < 0.001), which was neutralized by OIP5-AS1 overexpression (Figure 7d and e, *p* < 0.01), but was further enhanced by OIP5-AS1 knockdown (Figure 7d and e, *p* < 0.01). Collectively, miR-338-3p expression was upregulated in IL-1β-activated chondrocytes, and miR-338-3p was directly regulated by OIP5-AS1.

### 3.5 miR-338-3p reversed the regulatory effect of OIP5-AS1 on viability and apoptosis of IL-1β-activated chondrocytes

**Figure 8:**
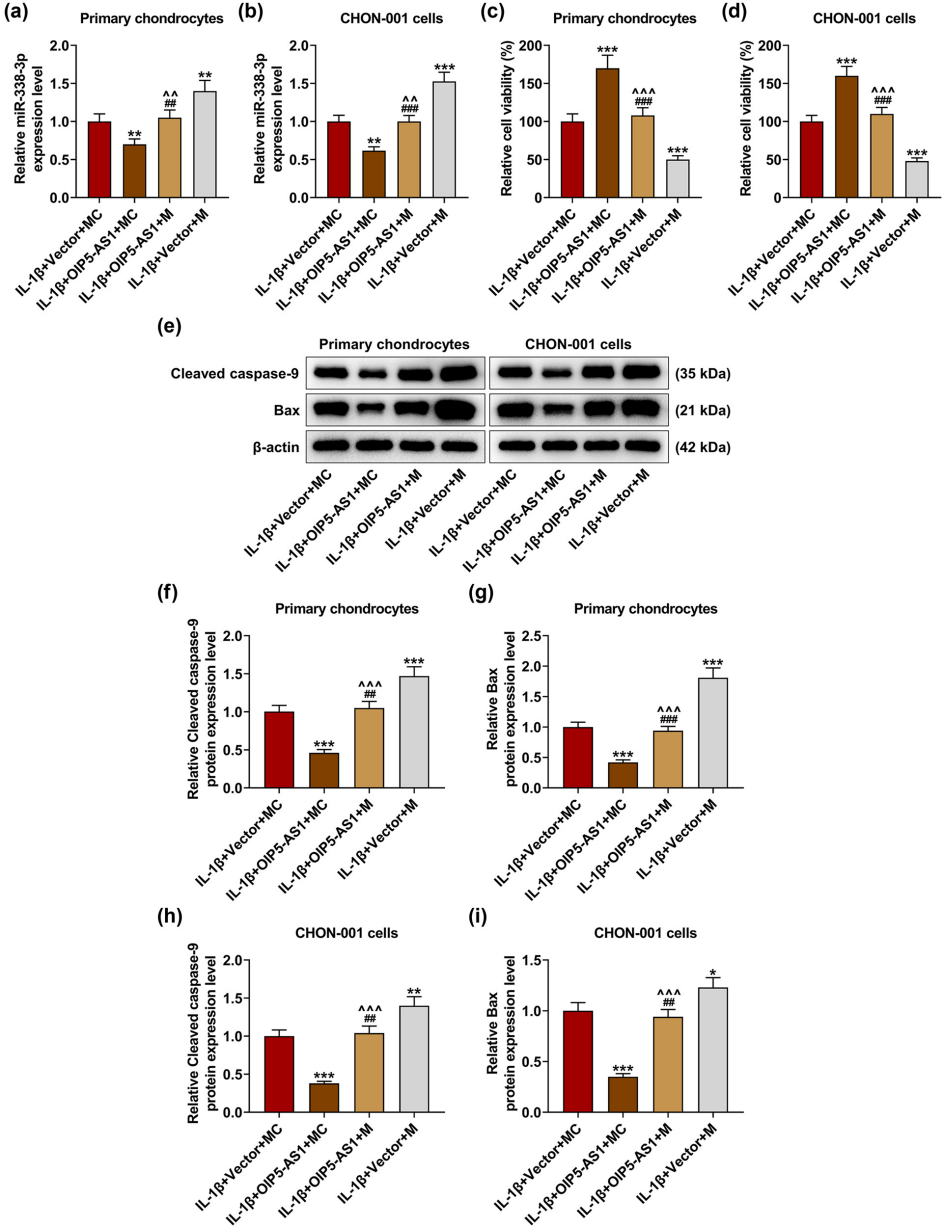
MiR-338-3p partially reversed the regulatory effects of OIP5-AS1 on viability and apoptosis of IL-1β-activated chondrocytes. Chondrocytes were co-transfected with miR-338-3p (M)/ MC and OIP5-AS1-overexpressing vector/empty vector, and exposed to IL-1β stimulation. (a–i) The level of miR-338-3p was examined using qRT-PCR, with U6 as internal control (a and b); cell viability was determined via cell counting kit-8 (CCK-8) assay (c and d); the protein levels of cleaved caspase-9 and Bax were detected using Western blot, with β-actin as internal control (e–i). IL-1β, interleukin-1β; MC, mimic control; qRT-PCR, quantitative reverse transcription-polymerase chain reaction. ^**^ *p* < 0.01 or ^***^ *p* < 0.001 vs IL-1β + vector + MC; ^^^^ *p* < 0.01 or ^^^^^ *p* < 0.001 vs IL-1β + OIP5-AS1 + MC; ^##^ *p* < 0.01 or ^###^ *p* < 0.001 vs IL-1β + vector + M.

As depicted in Figure 8a and b, the transfection of miR-338-3p mimic increased the level of miR-338-3p and partially neutralized the inhibitory effect of OIP5-AS1 overexpression on miR-338-3p level in IL-1β-activated chondrocytes (*p* < 0.01). miR-338-3p overexpression weakened viability and counteracted the promoting effect of OIP5-AS1 overexpression on the viability of IL-1β-activated chondrocytes (Figure 8c and d, *p* < 0.001). Furthermore, miR-338-3p upregulation augmented the levels of cleaved caspase-9 and Bax (Figure 8e–i, *p* < 0.01) and partially offset the inhibitory effects of OIP5-AS1 overexpression on these proteins in IL-1β-activated chondrocytes (Figure 8e–i, *p* < 0.001). In a word, OIP5-AS1 could mediate viability and apoptosis of IL-1β-activated chondrocytes by directly targeting miR-338-3p.

### 3.6 miR-338-3p attenuated the regulatory effects of OIP5-AS1 on ECM degradation and inflammation in IL-1β-activated chondrocytes

**Figure 9:**
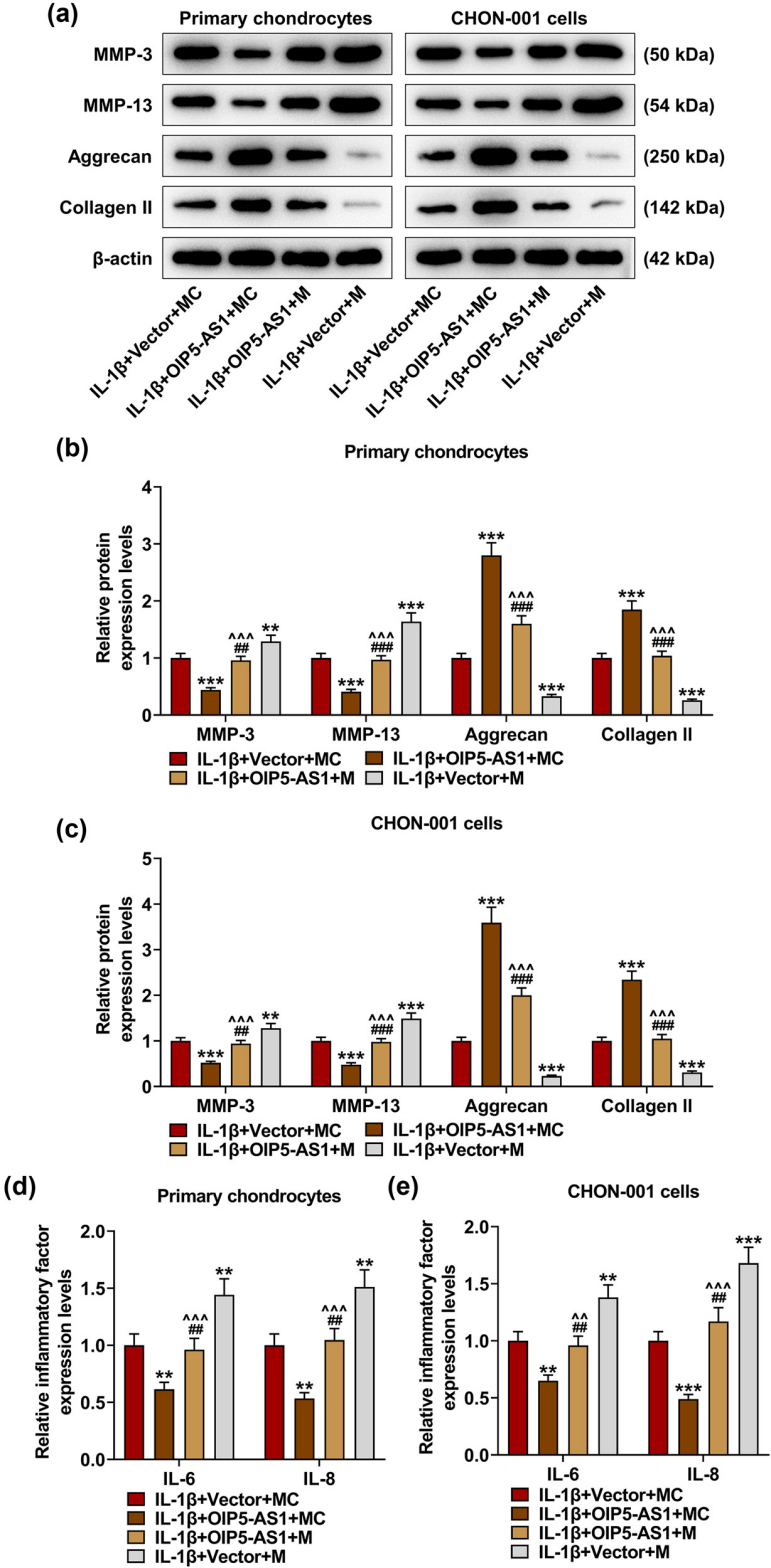
MiR-338-3p partially offset the regulatory effects of OIP5-AS1 on ECM degradation and inflammation of IL-1β-activated chondrocytes. Chondrocytes were co-transfected with miR-338-3p (M)/ MC and OIP5-AS1-overexpressing vector/empty vector, and exposed to IL-1β stimulation. (a–e) The levels of MMP-3, MMP-13, collagen II, and aggrecan were quantitated using Western blot (a–c), and the levels of inflammatory factors IL-6 and IL-8 were examined using qRT-PCR (d and e). IL-1β, interleukin-1β; MC, mimic control; MMP, matrix metalloproteinase; qRT-PCR, quantitative reverse transcription-polymerase chain reaction. ^**^ *p* < 0.01, ^***^ *p* < 0.001 vs IL-1β + vector + MC; ^^^^ *p* < 0.01, ^^^^^ *p* < 0.001 vs IL-1β + OIP5-AS1 + MC; ^##^ *p* < 0.01, ^###^ *p* < 0.001 vs IL-1β + vector + M.

miR-338-3p upregulation increased the protein levels of MMP-3 and MMP-13 as well as the mRNA levels of IL-6 and IL-8 (*p* < 0.001), while decreasing the levels of aggrecan and collagen II (*p* < 0.001). Moreover, miR-338-3p upregulation counteracted the effects of OIP5-AS1 overexpression on decreasing the levels of MMP-3, MMP-13, IL-6, and IL-8 and increasing the levels of aggrecan and collagen II (Figure 9a–e, *p* < 0.001). These findings indicated that OIP5-AS1 regulated ECM degradation and inflammation of IL-1β-activated chondrocytes by directly targeting miR-338-3p.

### 3.7 miR-338-3p offset the inhibitory effect of OIP5-AS1 on PI3K/AKT signaling activation

**Figure 10:**
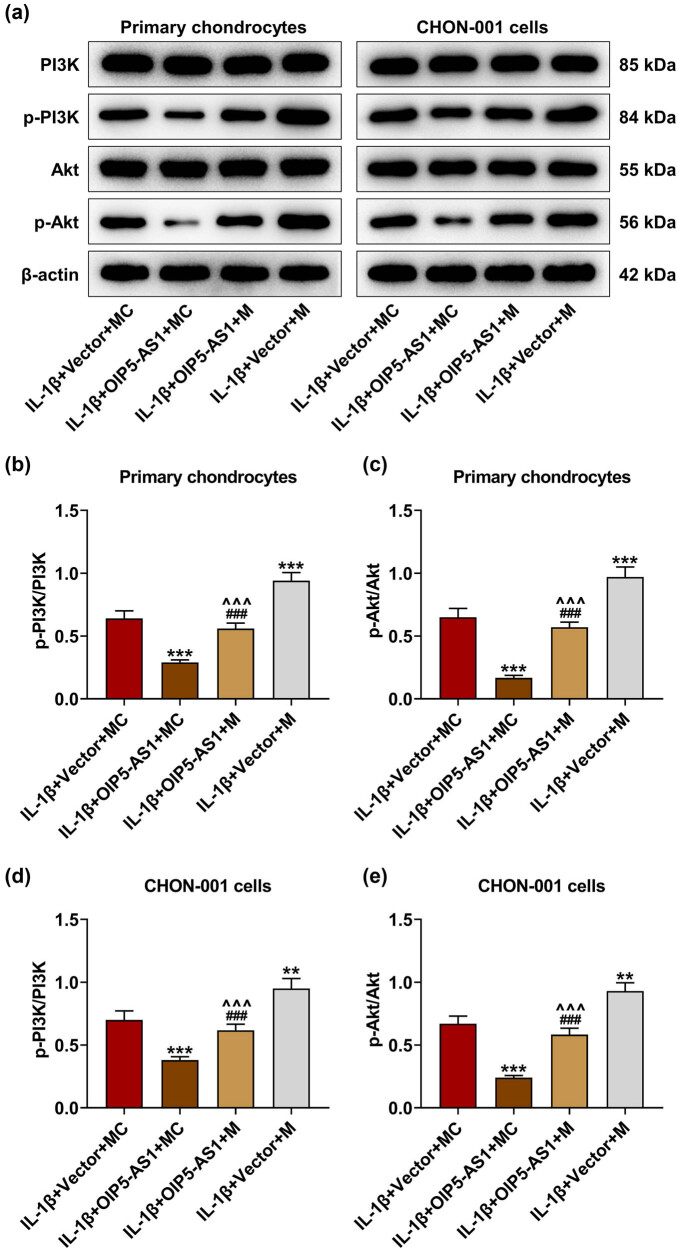
MiR-338-3p partially counteracted the inhibitory effect of OIP5-AS1 on PI3K/AKT signaling activation. Chondrocytes were transfected with miR-338-3p (M)/ MC and OIP5-AS1-overexpressing vector/empty vector, and exposed to IL-1β stimulation. (a–e) The protein levels of PI3K, phosphorylation (p)-PI3K, AKT, and p-AKT, as well as the ratios of p-PI3K/PI3K and p-AKT/AKT in primary chondrocytes and CHON-001 cells were examined using Western blot, with β-actin as internal control. ^**^ *p* < 0.01, ^***^ *p* < 0.001 vs IL-1β + vector + MC; ^^^^^ *p* < 0.001 vs IL-1β + OIP5-AS1 + MC; ^###^ *p* < 0.001 vs IL-1β + vector + M. IL-1β, interleukin-1β; MC, mimic control.

In OA progression, the PI3K/AKT pathway plays an important role in cell growth and survival [17]. OIP5-AS1 also regulates PI3K/AKT signaling activation by sponging miRNA [18]. Thus, PI3K/AKT signaling involved in this study was further explored. As shown in Figure 10a, the levels of PI3K and AKT did not change among all groups, whereas miR-338-3p overexpression increased the level of phosphorylation (p)-PI3K and p-AKT and partially counteracted the inhibitory effects of OIP5-AS1 overexpression on the levels of p-PI3K and p-AKT in IL-1β-activated chondrocytes (*p* < 0.001). In light of Figure 10b–e, miR-338-3p upregulation augmented the levels of p-PI3K/PI3K and p-AKT/AKT, and partially offset the inhibitory effects of OIP5-AS1 overexpression on the levels of p-PI3K/PI3K and p-AKT/AKT in IL-1β-activated chondrocytes (*p* < 0.001). These data demonstrated that OIP5-AS1 modulated PI3K/AKT signaling of IL-1β-activated chondrocytes by directly targeting miR-338-3p.

## 4 Discussion

In the present study, we found that the expression of OIP5-AS1 was downregulated in IL-1β-activated chondrocytes, while miR-338-3p was upregulated. OIP5-AS1 promotes viability and proliferation and inhibits apoptosis and ECM degradation in IL-1β-activated chondrocytes via targeting miR-338-3p by blocking the PI3K/AKT pathway.

During the initiation and progression of OA, the organization of the cartilage ECM is disrupted, which in turn damages the mechanical function of the organization. The OA severity is associated with the elevated levels of a series of pro-inflammatory factors [19]. Chondrocytes synthesize or degrade ECM via producing anabolic or catabolic factors [20]. In addition, promoting proliferation and inhibiting apoptosis in chondrocytes have been regarded as the key options to prevent or control the progression of OA [21]. As a consequence, exploring the role of critical OA-associated biomarkers in proliferation, apoptosis, ECM composition, as well as inflammation in chondrocytes might contribute to shedding new light on OA pathology and discovering novel avenues to prevent or control OA development.

The current findings showed that OIP5-AS1 was downregulated in IL-1β-treated chondrocytes, which was consistent with the previous results reported by Zhi et al. [8]. Furthermore, the biological function of OIP5-AS1 in IL-1β-stimulated chondrocytes was investigated. It was documented that OIP5-AS1 overexpression offset the effects of IL-1β on inhibiting cell viability, proliferation, and protein levels of aggrecan and collagen II, and promoting the apoptosis rate and protein levels of MMP-3, MMP-13, cleaved caspase-9, Bax, IL-6, and IL-8 in chondrocytes. However, OIP5-AS1 knockdown produced the opposite effects. The functional integrity of ECM, particularly rich in collagen II, is responsible for the function of normal articular cartilage [22]. Aggrecan is also the main structural ingredient of ECM. MMPs are the most important protease enzymes in ECM remodeling [23]. In OA, enzymes, such as MMP-3 and MMP-13, are increased in the joint space and indispensable for cartilage degradation [15]. MMP-13 is regarded as a primary contributor to cartilage degeneration of OA, because it preferentially cleaves collagen II [16]. In this study, OIP5-AS1 overexpression repressed IL-1β-stimulated viability decrease, apoptosis activation, ECM degradation, and inflammation enhancement in chondrocytes. Analogously, a previous report suggested that OIP5-AS1 overexpression drives the viability and suppresses the apoptosis and inflammation of chondrocytes [8].

The existing evidence unveiled that miRNA may have significant diagnostic and therapeutic potential and provide a novel option for OA treatment [24]. As per current findings, miR-338-3p expression is elevated in IL-1β-stimulated chondrocytes, implying miR-338-3p might have an association with OA pathology. Furthermore, we found that miR-338-3p was a direct target of OIP5-AS1 and inversely modulated by OIP5-AS1. To clarify the interplay of miR-338-3p and OIP5-AS1 in OA progression, we detected the roles of miR-338-3p and OIP5-AS1 in the viability, apoptosis, and inflammation in IL-1β-stimulated chondrocytes. The data proved that OIP5-AS1 promotes viability and inhibits apoptosis and ECM degradation in IL-1β-activated chondrocytes by directly regulating miR-338-3p.

In different biological processes, such as OA progression, the PI3K/AKT pathway plays a critical role in cell growth and survival [17]. It has been reported that AKT blocks pro-caspase-9 and phosphorylates caspase-9, thereby interfering with the apoptotic process [25]. Inactivation of PI3K/AKT/NF-κB pathway alleviates the inflammation and lessens the levels of MMP-3 and MMP-13 in human OA chondrocytes [26]. In the current work, we unraveled that OIP5-AS1 overexpression dampened the PI3K/AKT pathway by modulating miR-338-3p expression. Therefore, OIP5-AS1 may be dependent on blocking the PI3K/AKT pathway, so as to promote viability and inhibit apoptosis and ECM degradation by targeting miR-338-3p in IL-1β-activated chondrocytes. However, a limitation of this study is that since PI3K/AKT pathway is related to almost all cancers, exploring this one alone is far from enough to reveal the molecular mechanisms. Other potential molecular mechanisms still require further investigation.

Collectively, OIP5-AS1 promotes viability and proliferation and inhibits apoptosis and ECM degradation in IL-1β-activated chondrocytes by targeting miR-338-3p through blocking the PI3K/AKT pathway. The present results suggest that OIP5-AS1/miR-338-3p axis plays a regulatory role by blocking the PI3K/AKT signaling, which may be a new method for OA treatment. To better elucidate how OIP5-AS1/miR-338-3p axis ameliorates chondrocyte dysfunction by the PI3K/AKT signaling, PI3K/AKT inhibitors should be utilized in the future study, and more extensive experiments should be carried out in appropriate animal models for verification.
